# The Influence of Bovine Colostrum and Sodium Butyrate Supplementation on Gut Microbiota and the Intestinal–Liver Axis in Weaned Piglets

**DOI:** 10.3390/nu18111784

**Published:** 2026-06-01

**Authors:** Marek Pieszka, Kinga Szczepanik, Adam Lepczyński, Marta Marynowska, Maria Oczkowicz, Małgorzata Ożgo, Igor Łoniewski, Sylwia Orczewska-Dudek, Michalina Adaszyńska-Skwirzyńska, Bogdan Śliwinski, Karolina Skonieczna-Żydecka

**Affiliations:** 1 Department of Animal Nutrition and Feed Sciences, National Research Institute of Animal Production, Krakowska Str. 1, 32-083 Balice, Poland; marek.pieszka@iz.edu.pl (M.P.);; 2 Department of Physiology, Cytobiology and Proteomics, Faculty of Biotechnology and Animal Sciences, West Pomeranian University of Technology in Szczecin, Janickiego 29, 71-270 Szczecin, Poland; 3 Department of Animal Molecular Biology, National Research Institute of Animal Production, Krakowska Str. 1, 32-083 Balice, Poland; 4 Sanprobi sp. z o.o. sp. k., Kurza Stopka 5/C, 70-535 Szczecin, Poland; 5 Department of Biochemical Science, Pomeranian Medical University in Szczecin, 71-460 Szczecin, Poland; 6 Department of Monogastric Animal Sciences, Faculty of Biotechnology and Animal Sciences, West Pomeranian University of Technology in Szczecin, Janickiego 29, 71-270 Szczecin, Poland

**Keywords:** hepatointestinal communication, microbial diversity, short-chain fatty acids, pig nutrition, gut–liver axis

## Abstract

Dietary supplementation with sodium butyrate or bovine colostrum modulates the gut–liver axis in weaned piglets. Sodium butyrate exerted beneficial effects on liver function and lipid parameters, while also inhibiting inflammation and promoting the maintenance of the intestinal barrier. A particularly pronounced effect was observed with bovine colostrum supplementation, which significantly increased average daily weight gain (*p* < 0.001). In addition, piglets receiving colostrum consumed more feed and exhibited a significantly lower feed conversion ratio (*p* = 0.002). Metabolic changes induced by sodium butyrate and bovine colostrum supplementation resulted in alterations in the hepatic fatty acid profile, including a reduction in *n*-3 polyunsaturated fatty acids and a decrease in collagen fiber content in the liver (*p* = 0.03). The nutritional interventions did not significantly affect microbial diversity indices; however, marked changes in volatile fatty acid concentrations were observed in the large intestine. These changes indicate enhanced microbial fermentation and increased nutrient absorption in the experimental groups. Significant increases were detected in acetic acid (*p* = 0.003) as well as in butyric, isobutyric, and valeric acids (*p* = 0.014, *p* = 0.024, and *p* = 0.038, respectively). Supplementation with sodium butyrate and dried bovine colostrum also led to increased hepatic concentrations of macro- and microelements in piglets from the experimental groups. Genomic analyses suggest that sodium butyrate modulates hepatic metabolic and inflammatory pathways by downregulating *PPAR* (peroxisome proliferator-activated receptor) and *SIRT3* (sirtuin 3) expression and reducing *TNF* (tumor necrosis factor) gene expression, highlighting its potential role in regulating lipid metabolism, oxidative stress, and inflammation in a porcine model. Overall, the results indicate that both supplements may contribute to the modulation of gut microbial activity and liver metabolism in weaned piglets.

## 1. Introduction

Access to appropriate animal models is a *sine qua non* condition for developing safe preclinical protocols in biomedical research focused on humans. These models must enable the most objective verification of hypotheses and the acquisition of data that can be translated into human physiology. Domestic pigs occupy a special place in this hierarchy: the pig genome is approximately 94% identical to the human genome, and the structure and functioning of their internal organs, endocrine system, and digestive tract are very similar to those of humans. These characteristics predispose pigs to mimicking human inflammatory, metabolic, and neurodegenerative diseases [1,2].

In the present study, the model consisted of healthy post-weaning piglets, a critical developmental stage characterized by substantial stress, dietary transition, and intensive remodeling of the gut microbiota. The gastrointestinal tract is the largest interface between the organism and the external environment and serves as a highly selective barrier that ensures the controlled absorption of essential nutrients while limiting the translocation of harmful agents. The proper functioning of the intestinal barrier results from complex interactions between immune components, such as surface immunoglobulins and mucosal lymphocytes, and non-immune elements, including epithelial cells, tight junction proteins, and the mucus layer. The gut microbiota also plays a key role in maintaining intestinal homeostasis, while alterations in its composition may affect barrier function and intestinal integrity [3,4,5].

Throughout the 20th century, numerous studies addressed the issue of gut barrier function, and the topic remains highly relevant today. Despite significant advances in research methods, it is still unclear whether changes in intestinal barrier function are a primary cause of disease or develop as a result of chronic inflammation. Historically, bacteria were viewed mainly through the lens of their pathogenic representatives, with research focusing predominantly on their harmful effects. This perspective overshadowed the crucial role of commensal microorganisms in maintaining host health and supporting gut integrity [6,7].

Advances in microbiology have changed this view and shown that animals, including humans, are not separate biological entities but rather complex ecosystems. Their physiology depends on intricate interactions between host tissues and microbial communities, as well as among microbial species themselves. Bacteria inhabiting the gastrointestinal tract can be classified according to several criteria. One widely accepted classification distinguishes autochthonous (endogenous) bacteria, which are permanent gut residents capable of maintaining stable populations independently of food intake, from allochthonous bacteria, which are transient microorganisms primarily introduced through the diet. In pigs, this microbial ecosystem is particularly complex, comprising more than 500 different bacterial strains that play essential roles in maintaining intestinal homeostasis and overall health [8]. In humans, this number may be slightly higher, reaching about 1000 species [9]. By contrast, the total population of bacterial cells is estimated at 10^14^, which is ten times greater than the total number of cells in the human body [10], and the number of genes encoded by intestinal microorganisms reaches 2–4 million, roughly 100 times more than the host genome [9].

The species composition of indigenous bacteria varies across different segments of the pig gastrointestinal tract. This variation results from distinct physiological conditions that characterize each intestinal segment. Factors such as digesta transit time, the presence of specific digestive enzymes, hydrochloric acid, bile salts, nutrient availability, and oxygen concentration create diverse microenvironments that promote the proliferation of certain bacterial species while inhibiting others [11]. During the postnatal period, the gut microbiota of newborn piglets is dominated by genera such as *Bacteroides*, *Escherichia*, *Clostridium*, *Lactobacillus*, *Fusobacterium*, and *Prevotella*. As piglets mature, *Prevotella* and *Anaerovibrio* become predominant, while *Fusobacterium*, *Lactobacillus*, and various minor genera remain as secondary components during the post-weaning phase [12]. In the growth and fattening stages, the microbial community structure becomes relatively stable, with an increased abundance of fiber-degrading bacteria such as *Prevotella*, *Roseburia*, and *Clostridium*.

Weaning is a period of high stress for piglets and triggers dramatic changes in gut microbiota composition. The abundance of bacteria that utilize milk components decreases substantially, while species adapted to solid feed gradually dominate. Studies indicate that the symbiotic bacteria population directly or indirectly influences intestinal health in piglets, for example, by strengthening intestinal barrier function. After weaning, a decline in microbial diversity and in the abundance of “beneficial” bacteria is observed, alongside a relative increase in potentially pathogenic bacteria. These changes correlate with deteriorated health parameters and the reduced growth of piglets during adaptation to the new diet [13,14,15].

The results of studies conducted using high-throughput methods (sequencing, metabolomics) indicate a link between sodium butyrate in the intestines and the pathogenesis of inflammatory bowel disease and metabolic disorders. This, in turn, has underpinned the development of interventions aimed at modulating the microbiota, therapeutic strategies that include antibiotic therapy, probiotics, prebiotics, and other approaches to alter microbial composition. The clinical efficacy of these therapies remains limited and variable depending on the population and treatment protocol [16,17,18]. Their therapeutic effectiveness is, therefore, not ideal.

One of the active substances that influences intestinal epithelial cells is butyrate, a SCFA (short-chain fatty acid) produced by the microbial fermentation of dietary fiber. Butyrate exerts a range of beneficial effects on the intestinal epithelium that extend far beyond the colon. Butyrate serves as a fuel source for epithelial cells, has trophic effects on the intestinal mucosa, reduces intestinal inflammation, strengthens barrier function, stimulates the secretion of pancreatic and jejunal enzymes, modulates the ileal microbiota, and facilitates apoptosis of colon cancer cells [19,20]. Studies by Han et al. [21] demonstrated that butyrate administration reduced colonic inflammation by modulating the microbiota and metabolism along the gut–liver axis, suggesting the possible therapeutic mechanisms of butyrate. Microbiome analyses revealed a significant decrease in butyrate-producing bacteria in patients with IBD (inflammatory bowel disease) [22], and local administration of butyrate (colonic irrigation) alleviated inflammation in IBD models [23,24]. SCFAs also affect gastrointestinal motility. The increased contractility of colonic muscle after exposure to butyrate has been described in in vitro and ex vivo models [25,26,27]. In piglets, butyrate administration promoted the remodeling of the small intestinal mucosa, and parenteral supplementation with butyrate improved intestinal adaptation in a short bowel syndrome model [28,29,30].

Bovine colostrum is rich in bioactive factors (immunoglobulins, lysozyme, defensins, growth factors, protease inhibitors, and others [31]) that can accelerate gastrointestinal maturation, prime the immune system, and support the succession of a beneficial microbiota. In order for these factors to exert their physiological effects, they must reach their site of action in an active form. This process is aided by glycoproteins present in colostrum, protease inhibitors, and the relative resistance of certain components to digestion and degradation in an acidic environment. Some components of colostrum may also become activated during enzymatic digestion, resulting in the release of bioactive peptides. The antimicrobial effects of colostrum include the aggregation of microorganisms, neutralization of pathogens by immunoglobulins, and breakdown of bacterial cell walls through the action of lysozyme and defensins. Anti-inflammatory modulation may occur, among other ways, via inhibition of TLR4 (toll-like receptor 4) receptor activity on intestinal epithelial cells [32]. In vivo and in vitro studies indicate that supplementation with bovine colostrum improves intestinal structure and function, lowers levels of proinflammatory cytokines in the intestine (e.g., IL-1, IL-8), enhances weight gain, and supports neurological development in preterm piglet models [33,34,35,36].

Colostrum may also modulate bacterial populations through the direct inhibition of pathogens’ adhesion to epithelial cells (evidence from in vitro tests on cell lines) and the induction of epithelial protective mechanisms. Colostrum supplementation can affect hepatic trace element levels and growth parameters in piglets [37,38]. Those effects depend on the animals’ age, the colostrum dose, and its composition; the evidence in that field is still partly inconsistent and requires further studies [39].

The liver, as the central organ of metabolism and detoxification, is directly linked to the intestine via the portal venous circulation, which makes bacterial products and intestinal metabolites (including SCFAs) influential on hepatic functions. The gut–liver axis constitutes a system that connects the processing of intestinally derived products, regulation of metabolism, and maintenance of immunometabolic homeostasis. Consequently, changes in the gut microbiota and the metabolite profile can modulate liver functions, affecting energy metabolism and inflammatory status [40].

The research hypothesis assumes that sodium butyrate and bovine colostrum can change the shape of the intestinal microflora and alter metabolism in the gut–liver axis, which contributes to improved intestinal and liver health. The aim of this study was to evaluate the effect of adding dried bovine colostrum or sodium butyrate to the diet on the regulation of microflora and energy metabolism in the gut–liver axis in weaned piglets.

## 2. Materials and Methods

The experiment was conducted in the animal facility of the National Research Institute of Animal Production in the Department of Animal Nutrition and Feed Sciences, Balice, Poland.

### 2.1. Animals, Housing, Diet and Experimental Design

The experiment was conducted on 18 boars (2 × 9 piglets each) of the DanBred Hybrid breed at 28 days of age. The piglets were weighed on a laboratory scale to the nearest 0.01 kg and labeled. Their average initial piglet weight was 8.14 ± 0.33 kg. The piglets were then randomly assigned to 3 feeding groups with 6 individuals in each (*n* = 6). Piglets were placed in metabolic cages, one in each, and maintained under controlled environmental conditions at 20 °C and 60% humidity with a 12 h light/dark cycle. The cages were provided with feeders and teat feeders, and the animals were fed an amount of 2% of their body weight. Feed was given twice a day at 7 a.m. and at 3 p.m. From days 28 to 43, piglets received a weaning prestarter mix, and from day 43 to the end of the experiment, a starter mix. The composition of the feed mixtures is presented in Table S1 in the Supplementary Materials. The mixtures were prepared according to the standards of Pluske et al. [38]. From the beginning of the study, i.e., from day 28, the following additives were fed with the feed: group I, control—no additive, group II—dried bovine colostrum, and group III—sodium butyrate. The amount of feed intake was monitored daily. The piglets entered the experiment at 28 days of age and were slaughtered at 56 days of age following the experimental feeding period. In accordance with Polish law and European Union Directive 2010/63/EU, the study conducted as part of this research did not require approval from the Local Ethics Committee for Animal Experiments. All procedures complied with relevant guidelines concerning animal experimentation and the care of animals used in research. No procedures involving pain or suffering were performed. All analyses were carried out using post-mortem samples, and the slaughtering of animals solely for the purpose of obtaining organs or tissues is not classified as a procedure under the directive.

### 2.2. Biochemical Analyses

At animal slaughter, blood was collected into test tubes with a clotting activator (Equimed, Krakow, Poland) and centrifuged for 15 min and 4500 rpm, and the obtained serum was transferred to sterile Eppendorf-type tubes and placed at −80 °C until analysis in the laboratory. Analyses of selected biochemical parameters, including total cholesterol (CHOL), triglycerides (TG), albumin (ALB), alkaline phosphatase (ALP), alanine aminotransferase (ALT), aspartate aminotransferase (AST), and glucose (GLU), were performed by spectrophotometric method on a Mindray BS-180 automatic analyzer (Shenzhen Mindray Bio-medical Electronics Co., Ltd., Shenzhen, China), using dedicated diagnostic kits from PZ Cormay SA (Warsaw, Poland) following the manufacturer’s instructions.

### 2.3. Histology Analysis

A sample of liver, kidneys and pancreas tissue was removed from buffered formalin, cut into fragments of about 1 cm^2^ in size, and then placed in histology cassettes. The same anatomical regions were consistently collected from all animals, including a portion of the left lower lobe of the liver, while identical sampling sites were used for kidney and pancreas tissue collection. These cassettes were subsequently placed in a tissue processor, where they were dehydrated through increasing alcohol concentrations (30–100%). They were then cleared with xylene and embedded in paraffin. Two slides were produced from each block by slicing the blocks with a microtome HM 340E Electronic Rotary Microtome (Thermo Scientific, Waltham, MA, USA) into 4 μm sections. These liver preparations were stained using the Masson–Goldner trichrome technique with aniline blue to differentiate structures. Stained slides were viewed under an Olympus BX63 microscope (Olympus, Hamburg, Germany) equipped with an Olympus DP74 camera (all Olympus, Germany). The collected microscopic images underwent examination using Olympus cellSens Software and ImageJ (version 1.54; US National Institutes of Health, Bethesda, MD, USA; available at: http://rsodium.butytrate.info.nih.gov/ij/index.html, accessed on 24 June 2025). ImageJ software was used to calculate the percentage of collagen fibers (colored blue: collagen fibers), with a random selection of regions of interest (ROIs) from everyone (6 ROIs). Quantitative assessment of liver fibrosis, in this case specifically peritumoral fibrosis, depended on distinguishing colors between blue (collagen fibers) and red (parenchyma). The percentage of collagen fibers in the image was assessed using the Threshold function (after setting an 8-bit color scale). The percentage of collagen fibers was counted per area of ROI. Results were reported as averages for the individual.

Kidney and pancreas tissue sections were stained with hematoxylin and eosin (H&E). In the pancreas, the area of the islets of Langerhans was measured, and the number of constituent cells was counted. For each individual, 10 islets were analyzed. In the kidney, the number of glomeruli per 1 mm^2^ was determined by examining six regions of interest (ROIs), and the area of 15 randomly selected glomeruli was measured. For all parameters, values were first averaged per individual and then used to calculate group means.

All samples were appropriately blinded prior to analysis by means of coding.

### 2.4. Determination of Fatty Acid Profile on Liver

The analyses were carried out in accordance with PN-EN ISO 12966-2 [41]. Initially, sample preparation was performed. Frozen liver samples (−82 °C), each weighing approximately 1.0 g, were placed into 7.5 mL amber glass screw-cap vials with Teflon seals (Supelco, Bellefonte, PA, USA). To each vial, 5.0 mL of a 2:1 (*v*/*v*) chloroform:methanol mixture (Folch’s reagent) was added: chloroform (Chempur, Piekary Śląskie, Poland) and methanol (Merck, Darmstadt, Germany). The vials were sealed under nitrogen gas (Air-Liquide, Kraków, Poland) and shaken vigorously for 1 h using a Vibramax 100 laboratory shaker (Heidolph, Schwabach, Germany). After extraction, the vials were centrifuged at 2000 rpm for 20 min using a Z 205A centrifuge (Hermle, Gosheim, Germany) to separate the lipid-containing chloroform phase. This layer was transferred to 4.0 mL amber glass vials and sealed with Mininert^®^ Valves (both Supelco, USA) to allow derivatization under inert conditions. Chloroform was evaporated under a nitrogen stream, and 400.0 μL of 0.5 M KOH in methanol was added to the residue. The mixture was then heated at 80 °C for 30 min to hydrolyze lipids. Following hydrolysis, the samples were cooled to room temperature, and 500.0 μL of 14% boron trifluoride (BF_3_) in methanol (Sigma-Aldrich, St. Louis, MO, USA) was added for transesterification. The vials were incubated at 80 °C for another 30 min. After cooling, 1.0 mL of saturated NaCl solution and 2.0 mL of isooctane (POCH, Gliwice, Poland) were added to extract fatty acid methyl esters (FAMEs). The vials were shaken for 1 h and then left to stand for 30 min to separate phases. The isooctane layer was transferred to vials containing ~0.5 g of anhydrous sodium sulfate, then sealed under nitrogen. The dried FAME extracts were subjected to gas chromatography–mass spectrometry (GC-MS) using an Agilent 7820A GC coupled with a 5977E MSD (Agilent Technologies, Santa Clara, CA, USA) operating at 70 eV. Injections were made in split mode (5:1 ratio). Separation was achieved using an HP-5MS capillary column (30 m × 0.25 mm, 0.25 μm film thickness; 5% phenyl-methylpolysiloxane, Agilent Technologies). Helium was used as the carrier gas at a flow rate of 1 mL/min. The oven temperature was initially set to 80 °C (held for 2 min), then increased to 250 °C at a rate of 2 °C/min and held at the final temperature for 2 min. Injector- and transfer-line temperatures were maintained at 280 °C, while the ion source temperature was set at 230 °C. A solvent delay of 3 min was applied. The mass spectrometer scanned from 50 to 600 m/z, with a total run time of approximately 89 min per sample. Quantification was based on total ion current (TIC) peak areas using MSD ChemStation software (F.01.03.2357, Agilent Technologies). A standard mixture of 37 fatty acid methyl esters (FAME mix C4–C24, Sigma-Aldrich, St. Louis, MO, USA) was used for identification. All samples were analyzed in triplicate.

### 2.5. Analysis of Volatile Fatty Acids in Intestinal Contents

Samples for VFA (volatile fatty acid) determination were collected during dissection from the proximal colon, immediately transferred to scintillation vials, and frozen at −80 °C until analysis. FFA (free fatty acids) determination was performed using gas chromatography. Fatty acids from the sample (2 g) were extracted with H_2_O into a 50 mL flask. The solution was acidified with 24% sulfuric acid (5 mL of sample solution + 1 mL of acid solution). Volatile fatty acids analyses were performed using a Shimadzu QP2010Plus gas chromatograph equipped with a CP-Wax 58 capillary column (Shimadzu Corporation, Kyoto, Japan), (25 m × 0.53 mm, film thickness 1 µm). The injector and detector temperatures were set at 200 °C and 260 °C, respectively. Helium was used as the carrier gas at a flow rate of 5 mL/min. Quantitative calculations were carried out using the standard addition method.

### 2.6. The Concentrations of Selected Mineral Elements

The concentrations of selected mineral elements (P, K, Ca, Na, Mg, Zn, Si, Fe, Cu, Ba, Sr, Mn, Al, Se, Cr, Pb, Cd) were determined using inductively coupled plasma optical emission spectrometry (ICP-OES). Analyses were performed with an Optima 2000DV spectrometer (Perkin Elmer, Waltham, MA, USA) following sample mineralization in a Speedwave Xpert microwave digestion system (Berghof GmbH, Eningen, Germany), equipped with real-time temperature and pressure monitoring. For mineralization, 700.0 mg of frozen sample was accurately weighed into digestion vessels. Subsequently, 9.0 mL of 69% nitric acid (HNO_3_) and 1.0 mL of 30% hydrogen peroxide (H_2_O_2_), both from Supelco (Bellefonte, PA, USA), were added. The mixture was gently stirred and allowed to stand for 15 min before sealing the vessels and initiating the digestion process. Calibration curves were generated using TraceCert^®^ multi-element standard solutions (Sigma-Aldrich, St. Louis, MO, USA) at concentrations of 0.1, 0.5, 1.0, 2.0, 5.0, 10.0, 50.0, and 150.0 mg/L. Yttrium (Y) in HNO_3_ (Merck, Darmstadt, Germany) served as the internal standard. Before analysis, mineralized samples were diluted appropriately. A blank was prepared using demineralized water, 69% HNO_3_, yttrium solution, and a spectral buffer containing 1% Cs in 2% HCl. Calibration curves for all elements demonstrated strong linearity, with correlation coefficients (R^2^) exceeding 0.99. Average recovery rates ranged from 96% to 98%. All biological sample measurements were performed in triplicate. Quality control was ensured through the use of a certified reference material: Bovine Muscle 8414 (NIST, Gaithersodium butytrateurg, MD, USA).

### 2.7. Molecular Microbial Analysis of 16S rRNA of Fecal Samples

#### 2.7.1. Samples Collection

Fecal samples were collected from individual piglets from the distal colon after the animals were slaughtered. For each experimental group, 12 samples were collected before genomic DNA extraction. Feces were collected in sterile containers and stored at −80 °C until analyzed.

#### 2.7.2. DNA Isolation

DNA was extracted from each sample using the ZymoBiomics DNA Miniprep Kit (Zymo Research, Los Angeles, CA, USA) following the manufacturer’s protocol. DNA concentration was quantified using a Qubit 2.0 fluorometer (Invitrogen, San Diego, CA, USA). To monitor for potential contamination during the extraction process, a negative control consisting of Milli-Q water was included and processed alongside the test samples under identical conditions. This control was used during downstream analysis to assess laboratory-derived contamination.

Bioinformatic processing of the sequencing data included the use of the decontam package in R to identify and eliminate potential contaminants. This quality control step enhanced the reliability of results related to the bacterial community composition, as determined by analysis of the V3 hypervariable region of the 16S rRNA gene and metagenomic sequencing data.

#### 2.7.3. Amplification of the 16S rRNA Gene

To study the intestinal microbiome of piglets using sequencing on the Ion Torrent PGM platform, the hypervariable V3 region of the 16S rRNA gene was selected. Universal primers 337F and 518R were used for amplification of bacterial DNA. Amplification was performed using the FastStart SYBR Green Master kit (Basel, Switzerland) in the following scheme: 94 °C for 4 min; 37 cycles of 94 °C for 30 s, 53 °C for 30 s, and 72 °C for 30 s; and final extension at 72 °C for 5 min.

### 2.8. RNA Isolation and Quantitative PCR

RNA isolation from liver tissue was conducted using the Total RNA Mini (A&A Biotechnology, Gdańsk, Poland) following the producer’s recommendations. The RNA quality was assessed using the Tapestation 2200 (Agilent, Santa Clara, CA, USA), while its quantity was measured with the Nanodrop 2200 (Thermofisher Scientific, Waltham, MA, USA). The RNA quality was assessed by agarose gel electrophoresis. The RNA was then reverse-transcribed using the High Capacity cDNA Archive Kit (Thermofisher Scientific, Waltham, MA, USA). Subsequently, qPCR was performed using TaqMan Gene Expression Assays: *TNF* ss03391318, *PPAR* ss03380164, *ACADM* ss03393699, *CAT* ss04323025, *NOTCH1* Ss03377164, *SIRT3* Ss03386766, *SOD2* ss03374828, *GSS* ss04328106 (Thermofisher Scientific, Waltham, MA, USA). The process was performed in triplicate on the QuantStudio 7-flex instrument (Thermofisher Scientific, Waltham, MA, USA) using the TaqMan Gene Expression Master Mix (Thermofisher Scientific, Waltham, MA, USA). For an endogenous control, *RPL27* Ss3385714_g1 was used.

### 2.9. Statistical Analysis

Data on biochemical, morphometric, and histometric analyses and results of concentrations of selected mineral elements were analyzed using one-way ANOVA. Results are presented as means and corresponding standard SEM (error of the mean). Before data analysis, normality was checked using the Shapiro–Wilk test, and histograms were evaluated. The homogeneity of variances was assessed using Levene’s test. The Tukey post hoc test with correction for multiple comparisons was used to compare differences between means when the overall effect was considered significant (*p* < 0.05). All analyses were performed using the software package Statistica^®^ ver. 13.3 (StatSoft Inc., Tulsa, OK, USA).

In the absence of a normal distribution, the non-parametric Kruskal–Wallis test was applied (results of liver fatty acid profile analysis and gene expression). When significant differences were detected, pairwise comparisons were performed using Dunn’s post hoc test with Bonferroni correction. Statistical significance was set at *p* < 0.05.

#### Statistical Analysis of the Microbiota

Statistical analysis was conducted utilizing R statistical software (version 4.2.2 [42]). Alpha diversity metrics were computed after rarefaction at a depth of 88.175 utilizing the rtk 0.2.6.1 package [43]. The calculated metrics included Richness, Evenness, Simpson, and Shannon indices. To discern significant differences among groups, the non-parametric Kruskal–Wallis test was employed.

For beta diversity assessment, the Bray–Curtis dissimilarity metric was computed from rarefied data employing the vegan (version 2.6-4 [44]) package. Differences between analyzed groups were evaluated using the PERMANOVA test. Differential Abundance Analysis- rarefied abundance data underwent transformation into relative abundances. Mean values across study groups were compared utilizing the Kruskal–Wallis test. Visualization of results was executed through the generation of a Manhattan plot, plotting the adjusted -log10(*p*-value) against the features. Within the plot, distinct -log10(*p*-value) threshold was highlighted for significance determination (green color).

## 3. Results

All animals included in the experiment remained clinically healthy throughout the study period and showed no visible signs of disease. The results regarding zootechnical parameters, including weight gain, feed intake, and animal weight, were published in an earlier paper by Pieszka et al. [45].

### 3.1. Biochemistry Analysis

No statistically significant differences were observed in CHOL, TG, ALT, AST, ALP, GLU, or ALB levels (Table 1).

### 3.2. Histological Analysis

No statistically significant differences were observed in the kidney and pancreas structure. However, a significant reduction in the percentage of connective tissue in the liver was observed in group III compared to the other groups (*p* = 0.030). The results are presented in Table 2 and Supplementary Figure S1.

### 3.3. Microbiota Analysis

Alpha Diversity: No statistically significant differences in alpha diversity metrics—richness, evenness, Simpson, and Shannon indices (Supplementary Figure S2)—were observed between study groups (all *p* > 0.05).

Beta Diversity: No significant differences in beta diversity, as measured by Bray–Curtis dissimilarity, were detected between groups (Supplementary Figure S3, *p* > 0.05).

Differential Abundance Analysis: Comparisons of mean feature values across groups revealed no significant differences in differential abundance for any features, as shown in the Manhattan plot (Supplementary Figure S4, all *p* > 0.05, -log10(0.05) = 1.3).

### 3.4. Analysis of Fatty Acid Profile in the Liver

Significant differences were observed in cis-8,11,14-eicosatrienoic acid (*p* = 0.032), with group III showing higher levels compared to group II. Total polyunsaturated fatty acids (PUFAs) also differed significantly among groups (*p* = 0.042), with group I having higher values than group II. Likewise, ω-3 fatty acids were significantly higher in group I compared to group II (*p* = 0.034). A tendency toward differences was observed for cis-11,14,17-eicosatrienoic acid (*p* = 0.082), cis-5,8,11,14,17-eicosapentaenoic acid (*p* = 0.086), and the PUFA/SFA ratio (*p* = 0.051), suggesting numerically higher values in groups I and III compared to group II. No statistically significant differences were observed among groups I, II, and III in the concentrations of most fatty acids, including myristic acid, palmitic acid, palmitoleic acid, heptadecanoic acid, cis-10-heptadecenoic acid, stearic acid, oleic acid, linoleic acid, γ-linolenic acid, α-linolenic acid, cis-11-eicosenoic acid, cis-11,14-eicosadienoic acid, arachidic acid, and nervonic acid. Similarly, no significant differences were found in total saturated fatty acids (SFAs), monounsaturated fatty acids (MUFAs), ω-6 fatty acids, MUFA/SFA, MUFA/PUFA, or ω-6/ω-3 ratio. All results are presented in Table 3.

### 3.5. Results of the Concentration of Selected Mineral Elements

Significant differences were observed in several mineral concentrations among the experimental groups (Table 4). Group II showed significantly higher magnesium (Mg) levels compared to groups I and III (*p* = 0.0001). Calcium (Ca) concentration was significantly lower in group I compared to groups II and III (*p* = 0.009). Sodium (Na) content differed among groups (*p* = 0.032), with group III presenting the highest value and group I the lowest. Potassium (K) levels were significantly higher in group II compared to groups I and III (*p* = 0.006). Copper (Cu) was also markedly elevated in groups II and III compared to group I (*p* = 0.002). Iron (Fe) concentration was highest in group II and lowest in group III, with significant differences across all groups (*p* = 0.0001). Manganese (Mn) content differed significantly among groups (*p* = 0.041), with group III presenting the lowest value. However, phosphorus (P) content differed significantly among groups (*p* = 0.0002), with group II having the highest value and group III the lowest. Zinc (Zn) showed a tendency toward differences (*p* = 0.076), with group III exhibiting numerically higher levels compared to group II. No significant differences were found for chromium (Cr), lead (Pb), cadmium (Cd), selenium (Se), or aluminum (Al).

### 3.6. Results of Gene Expression Analysis in the Liver

In group III, a significant reduction in *PPAR* expression was observed compared to group I (*p* = 0.006). Several genes showed a tendency toward differential expression. Group III showed a tendency toward lower *TNF* expression than group II (*p* = 0.082). A tendency toward reduced expression was observed for *SIRT3* in group III compared to group I (*p* = 0.082). No statistically significant differences in the expression of *ACADM*, *CAT*, *NOTCH1*, or *SOD2* were observed between the groups. The results are presented in Table 5.

### 3.7. Results of the Average Content of Short-Chain Fatty Acids (SCFA) in the Large Intestine Content of Piglets at 56 Days of Age (µmol/g Wet Weight)

The volatile fatty acid (VFA) profile was determined in the intestinal contents of piglets (Table 6). The addition of bovine colostrum and sodium butyrate to the diet resulted in an increase in acetic acid content (*p* = 0.003). In the case of propionic acid, no significant changes in the level of this acid were observed between the groups (*p* = 0.258). However, in the case of butyric, isobutyric and valeric acids, as in the case of acetic acid, a significant increase in the levels of these acids was found in the experimental groups compared to the control group, respectively: *p* = 0.014; *p* = 0.024; and *p* = 0.038.

## 4. Discussion

The weaning period is critical for piglets, as stress and the change in diet from milk to solid feed lead to malnutrition, decreased immunity, and changes in the composition of the intestinal microbiota [46,47]. After weaning, the diversity and abundance of beneficial bacteria decrease, while the abundance of pathogenic bacteria increases, which affects the functioning of the intestinal barrier and the maturation of immunity [15,48]. The gut microbiome is a key component of the gut–liver axis, and its disruption can lead to the penetration of bacterial products into the portal circulation and cause liver damage [49,50]. The gut–liver axis regulates metabolism, processes intestinal products, and maintains immune homeostasis, which highlights the importance of maintaining a healthy gut microbiome for the entire organism [40].

In order to improve health during and immediately after weaning, nutritional supplements that support the digestive system are used. A study by Poulsen et al. [51] showed that administering colostrum to piglets immediately after weaning reduced the number of potentially pathogenic *Escherichia coli* in intestinal contents and feces compared to piglets fed milk replacer, suggesting a strong influence of the colostrum on the composition of the microbiome. Research by Huang et al. [52] showed the effect of sodium butyrate on changes in the colon microbiota, especially in the case of an increase in the relative abundance of *Bacteroidetes*, *Ruminococcaceae*, and *Lachnospiraceae* in the colon of piglets. This may enhance the host’s ability to obtain more energy from complex polysaccharides that are resistant to digestive enzymes. It should be noted that differences between the present findings and those reported in other studies may also reflect variability in pig genotype, age, diet composition, and other experimental conditions, which should be considered when interpreting the results. However, in the current experiment, administration of sodium butyrate and colostrum did not result in statistically significant differences in the overall composition of the gut microbiota.

An interesting result is the reduction in the percentage of connective tissue—collagen fibers—in the liver of piglets receiving sodium butyrate. This is reflected in studies that have found butyrate to have a hepatoprotective effect on the liver. It has been found that the anti-fibrotic effects can be partly attributed to the direct effect of butyrate on collagen production in hepatic stellate cells, including the inhibition of non-canonical TGF-β signaling pathways [53,54]. The results of another study by Zhao et al. [55] showed that butyrate can inhibit liver fibrosis in infants with biliary atresia by downregulating *HDAC2* expression and modulation of the *IL-6/STAT3* pathway, and it can improve the structure of the gut microbiota, which may promote liver function preservation. The experiment did not reveal any significant differences in biochemical parameters related to liver function (CHOL, TG, ALT, AST, GLU, ALB, ALP), but it should be noted that all animals were healthy and the parameters were within the reference ranges [56]. Therefore, given that butyrate reduced the percentage of collagen in liver tissue without altering the main serum liver markers, it can be suggested that butyrate may influence hepatic extracellular matrix remodeling without detectable effects on the standard biochemical indicators of liver function under the conditions of this study.

*PPAR* is a receptor that plays a key role in fatty acid oxidation and lipid and lipoprotein metabolism, as well as in the regulation of inflammation and oxidative stress [57]. Its central role in energy balance, lipid metabolism, and inflammatory processes makes it a crucial factor in the development of obesity-related diseases and a promising therapeutic target for metabolic disorders. The expression and action of *PPARα* vary between species. In rodents, *PPARα* is strongly expressed, and its activation induces genes related to lipid metabolism and leads to the proliferation of peroxisomes in the liver [58]. In species such as pigs and humans, *PPARα* activation does not show this effect due to lower expression and weaker gene response [59]. In this experiment, the downregulation of *PPAR*αgene expression under the influence of colostrum was observed. In the pig model, *PPARα* activation correlates with the increased expression of β-oxidation genes and better utilization of fats from colostrum, while decreased *PPARα* activity or expression leads to reduced fatty acid oxidation and a tendency to accumulate lipids in the liver [60,61,62].

The experiment revealed a tendency for decreased expression of the *SIRT3* gene in the sodium butyrate group compared to the control group. *SIRT3* is located in the mitochondrial matrix and is responsible for the regulation of metabolic enzymes’ acetylation level, including acetyl-coenzyme A synthetase. *SIRT3* plays a key role in complex diseases such as metabolic syndrome, diabetes, and NAFLD. Furthermore, evidence from rodent models indicates a link between SIRT3 activity, gut microbiota alterations, and hepatic lipid metabolism, with emerging data suggesting similar associations in pigs [63,64]. Studies have shown that a high-fat diet downregulates *SIRT3* in the liver. Because SIRT3 is a key mitochondrial deacetylase that regulates enzymes involved in fatty acid oxidation and antioxidant defense, its depletion leads to increased mitochondrial ROS production. This oxidative stress disrupts mitochondrial integrity and amplifies lipid-induced hepatocellular injury, thereby exacerbating lipotoxicity. It has also been shown that *SIRT3* deficiency increases susceptibility to direct oxidative stress and facilitates ROS-dependent oncogenic transformation [65].

In the current experiment, a beneficial effect of sodium butyrate on the reduction of *TNF* expression in liver tissue was observed. This finding is consistent with previously published studies indicating that sodium butyrate can downregulate the expression of several pro-inflammatory cytokines and mediators [66,67,68]. Such anti-inflammatory properties suggest that sodium butyrate may play a meaningful role in modulating immune responses and reducing the risk of inflammation-related metabolic disturbances. Collectively, these results support the potential of sodium butyrate as a functional dietary additive capable of contributing to the prevention or attenuation of inflammatory processes.

SCFAs are characterized by carbon atom chains with fewer than six carbon atoms and consist mainly of acetate, propionate, and butyrate [69]. These SCFAs are the main end products of microbial fermentation in the colon and serve as the major anions in the mammalian large intestine [70]. The rate of production and distribution of SCFAs largely depends on the diversity and abundance of the colonic gut microbiota, as well as the type of substrate and intestinal transit time [71]. The species primarily responsible for SCFA synthesis include *Eubacterium*, *Roseburia*, *Clostridium* anaerobes, *Streptococcus*, *Bacteroides*, and *Bifidobacterium* [72]. Acetate is mainly produced by *Bifidobacterium*, *Lactobacillus* and *Heprevotella*. Propionate, on the other hand, is mainly produced by *Bacteroides* and *Firmicutes*. Butyrate, on the other hand, is mainly the result of fermentation by *Firmicutes*, especially species from the *Verrucomicrobiaceae* and *Lachnospiraceae* families [73,74]. In this study, we found changes in the content of microbial metabolites, with an increase in acetic acid levels parallel to an increase in propionic and butyric acids in the colon of piglets receiving supplementation. This increase in acetic, propionic, and butyric acid content may be an effect of the selective promotion of acetic and propionic bacteria activity, conversion of butyric acid, and supplementation of sodium butyrate to acetic acid by those bacteria groups [75]. Among SCFAs, the production of butyrate by specific gut microbiota plays a key role in the promotion of growth and development of both the large and small intestines [76]. Therefore, it appears that the addition of sodium butyrate or bovine colostrum is able to modulate the number of microorganisms and fermentation in the large intestine. SCFAs’ function ranges from being a source of energy to being a key mediator of immune cell function. SCFAs can also induce liver metabolism, strengthen intestinal barrier function, and alleviate symptoms associated with IBD [77,78]. Thus, SCFAs may play an important role in IBD therapy by regulating the gut–liver axis [79].

The conducted study revealed significant changes in the fatty acid composition of the livers of animals supplemented with bovine colostrum, compared to the control group. In this group of animals, a decrease in the total PUFA content in this organ was observed, which reflects a decrease in the content of their *n*-3 fraction. This is probably the result of higher SFA intake with bovine colostrum. Supplementation with 20 g of dried bovine colostrum per day provided approximately 0.94 g of additional SFA, considering that bovine colostrum contains approximately 6.7% fat, of which about 70% is saturated fatty acids [80,81]. Bovine colostrum is a rich source of saturated and monounsaturated fatty acids. The main fatty acids in bovine colostrum include palmitic, oleic, and myristic acids [82,83]. The increase in the SFA and MUFA (total of ~0.3%) content in the livers of animals supplemented with colostrum, although not statistically significant, is offset by a significant reduction in the overall PUFA content. Therefore, it appears that the enrichment of the grain-based diet of the piglets with dried bovine colostrum may have caused changes in the relative proportions of fatty acids in the liver, as a primary site of their metabolism after absorption from the gastrointestinal tract. Supplementation with both bovine colostrum and sodium butyrate significantly affected the macro- and micronutrient content in the liver. Supplementation with a colostrum-containing preparation resulted in an increase in the Ca, Mg, Fe, Cu, and K content in this organ when compared to the control group. This is not surprising, as bovine colostrum is a rich source of Ca, Mg, Fe, Cu, P, and K [84]. A number of mechanisms related to the regulatory role of colostrum in calcium absorption have been identified to date. In addition to being a rich source of this element, colostrum also contains vitamin D and bioactive proteins derived from colostrum, which may contribute to the regulation of its absorption and metabolism [84,85]. The absorption of iron from colostrum, mainly in the form incorporated into the lactoferrin molecule [86], is an effective process, and its efficiency is comparable to the ability to absorb iron in inorganic form as FeSO_4_ [87]. Moreover, supplementation with lactoferrin significantly supports the supplementation of inorganic iron [88]. It is a synergistic effect related to other mechanisms responsible for the absorption of iron in organic form. It has to also be stated that this route of lactoferrin absorption is also effective in piglets in the post-weaning period [89,90]. Apart from the well-established high levels of magnesium, copper, and potassium in colostrum, which may enhance their absorption from the gastrointestinal tract, no specific mechanisms facilitating the uptake of these elements have been identified, unlike those described for calcium and iron.

As mentioned above, the use of sodium butyrate as a dietary supplement for growing pigs has also significantly affected the liver mineral content. Compared to the control group, the livers of pigs supplemented with sodium butyrate (1 g/day/pig) had higher Na, Ca, and Cu levels and lower Fe content. The higher sodium content in supplemented animals is primarily due to the use of sodium butyrate. In addition, it has been shown that butyric acid can stimulate the expression/activity of ion transporters in the large intestine, including the epithelial sodium channel, which is responsible for sodium absorption from the gastrointestinal tract [91]. Although butyrate is assumed to act in the colon, its actual delivery to the large intestine may be limited. A similar mechanism is associated with increased calcium absorption. In a mouse model, it was shown that sodium butyrate supplementation caused an increase in the expression of the epithelial Ca^2+^ channel (Transient Receptor Potential Vanilloid subfamily member 6—TRPV6) in the colon, which resulted in increased calcium absorption from the gastrointestinal tract [92]. We assumed a similar mechanism based on our previous studies investigating dietary supplementation with ITF (inulin-type fructans), whose fermentation by gut microbiota increases SCFA production and may consequently stimulate the absorption of electrolytes, including sodium and calcium [93,94]. ITF supplementation also appears to stimulate the bone formation process, as reflected in the increased activity of bone formation markers [95]. Sodium butyrate supplementation may have a significant impact on iron absorption from the gastrointestinal tract, as well as the metabolism of this element in the body. Sodium butyrate may limit iron absorption in the small intestine by regulating the expression of its transporters in the EPAS1 transcription factor-dependent pathway [96]. On the other hand, the absorption of iron ions in the distal sections of the gastrointestinal tract may be promoted by a reduction in the pH of the gastrointestinal contents by SCFAs, including butyric acid [97]. It should be mentioned that iron metabolism and distribution in the body can also be regulated by sodium butyrate. It has been shown that sodium butyrate supplementation regulates, among other things, the response that promotes the reduction of inflammation-regulated anemia by facilitating the export of iron from macrophages through the stimulation of ferroprotein expression and distribution [98]. It has also been shown that iron levels in hepatocytes can be regulated by sodium butyrate in order to reduce ferroptosis in the event of metabolic disorders. These phenomena are directly dependent on the health status, iron status, and composition of the gut microbiota. It, therefore, appears that the reduction in iron concentration in the liver of piglets supplemented with sodium butyrate may be the result of the regulatory effect of sodium butyrate on many mechanisms related, among other things, to its absorption and inflammatory response, thus affecting its tissue distribution, especially in growing animals during the weaning period. It can also be assumed that the high Cu content in the liver compared to the control group may be the result of increased absorption in the large intestine due to increased solubility in acidified digestive contents.

## 5. Conclusions

In conclusion, supplementation with sodium butyrate and bovine colostrum modulated parameters associated with the gut–liver axis in weaned piglets. These effects were reflected in changes in gut microbiota-related metabolites, including increased SCFA (short-chain fatty acid) concentrations, as well as alterations in hepatic gene expression related to inflammatory and metabolic pathways. Sodium butyrate supplementation was additionally associated with a reduction in connective tissue content in the liver, which suggests potential effects on hepatic tissue structure. Overall, the results indicate that both supplements may contribute to the modulation of gut microbial activity and liver metabolism in weaned piglets.

## Figures and Tables

**Table 1:** Effect of colostrum or sodium butyrate on biochemical parameters of piglets.

	Nutrition Group				
	I	II	III	SEM	*p*-Value
Number of piglets, no	6	6	6		
Biochemical parameters					
CHOL, mg/dL	77.750	77.083	62.333	4.77	0.349
TG, mg/dL	37.255	45.548	40.268	3.56	0.656
ALT, U/L	82.292	78.108	70.183	13.08	0.646
AST, U/L	84.971	79.516	106.661	5.12	0.686
GLU, mg/dL	110.750	109.833	92.750	5.26	0.308
ALB, g/dL	2.786	2.619	2.407	0.19	0.732
ALP, U/L	290.667	349.000	269.583	18.60	0.202

I—control group, no additive; II—group with dried bovine colostrum; III—group with sodium butyrate. CHOL—total cholesterol, TG—triacylglycerides, ALT—alanine aminotransferase, AST—aspartate aminotransferase, ALP—alkaline phosphatase, GLU—glucose, ALB—albumin. SEM: error of the mean.

**Table 2:** Effect of colostrum or sodium butyrate on the results of histomorphometric analyses of piglet kidney, liver and pancreas.

	Nutrition Group				
	I	II	III	SEM	*p*-Value
Number of piglets, no	6	6	6		
Kidneys					
Surface area of glomurus (µm^2^)	2665.900	3404.475	2665.271	112.93	0.268
Number of renal corpuscles per mm^2^	13.924	12.610	14.711	0.70	0.750
Pancreas					
Surface area of pancreatic follicles (µm^2^)	6896.659	5822.421	4565.054	964.94	0.677
Number of follicular cells per pancreatic follicle	113.000	80.922	92.333	13.15	0.664
Liver					
Percentage of collagen fibers (%)	2.026 ^a^	2.068 ^a^	1.316 ^b^	0.10	0.030

I—control group, no additive; II—group with dried bovine colostrum; III—group with sodium butyrate. ^a,b^—Values within a row with different superscripts differ significantly at *p* < 0.05. SEM: error of the mean.

**Table 3:** Effect of colostrum or sodium butyrate on liver fatty acid profile.

	Nutrition			
	Mean ± SD			*p*-Value
	**I**	**II**	**III**	
N experimental units	6	6	6	
Myrystic acid	1.112 ± 0.01	1.118 ± 0.01	1.128 ± 0.02	0.201
Palmitic acid	16.497 ± 0.40	16.585 ± 0.29	16.583 ± 0.37	0.850
Palmitoleic acid	1.933 ± 0.15	1.970 ± 0.13	2.012 ± 0.08	0.873
Heptadecanoic acid	0.806 ± 0.05	0.860 ± 0.06	0.837 ± 0.06	0.482
cis-10-heptadecenoic acid	0.147 ± 0.02	0.158 ± 0.02	0.158 ± 0.01	0.804
Stearic acid	30.448 ± 0.45	30.450 ± 0.30	30.258 ± 0.41	0.665
Oleic acid	21.367 ± 0.47	21.545 ± 0.35	21.380 ± 0.50	0.653
Linoleic acid	12.132 ± 0.30	11.997 ± 0.42	12.292 ± 0.36	0.444
γ-linolenic acid	0.069 ± 0.01	0.072 ± 0.00	0.068 ± 0.00	0.514
α-linolenic acid	0.322 ± 0.04	0.293 ± 0.01	0.320 ± 0.03	0.135
cis-11-eicosenoic acid	0.061 ± 0.00	0.060 ± 0.00	0.060 ± 0.00	0.367
cis-11,14-eicosadienoic acid	0.791 ± 0.10	0.728 ± 0.10	0.717 ± 0.08	0.288
cis-8,11,14-eicosatrienoic acid	0.433 ± 0.03 ^ab^	0.375 ± 0.05 ^a^	0.445 ± 0.04 ^b^	0.032
cis-11,14,17-eicosatrienoic acid	0.599 ± 0.01 ^de^	0.547 ± 0.03 ^d^	0.577 ± 0.06 ^e^	0.082
Arachidic acid	12.978 ± 0.17	12.943 ± 0.19	12.868 ± 0.13	0.706
cis-5,8,11,14,17-eicosapentanoic acid	0.195 ± 0.01 ^d^	0.187 ± 0.01 ^de^	0.185 ± 0.01 ^e^	0.086
Nervonic acid	0.109 ± 0.01	0.112 ± 0.00	0.112 ± 0.00	0.812
SFA	48.863 ± 0.51	49.013 ± 0.11	48.807 ± 0.40	0.548
MUFA	23.508 ± 0.61	23.733 ± 0.27	23.610 ± 0.45	0.728
PUFA	27.518 ± 0.36 ^a^	27.142 ± 0.22 ^b^	27.472 ± 0.30 ^ab^	0.042
*ω*3	1.115 ± 0.04 ^a^	1.027 ± 0.03 ^b^	1.082 ± 0.08 ^ab^	0.034
*ω*6	26.403 ± 0.38	26.115 ± 0.21	26.390 ± 0.25	0.142
MUFA/SFA	0.481 ± 0.02	0.484 ± 0.01	0.484 ± 0.01	0.796
MUFA/PUFA	0.855 ± 0.03	0.875 ± 0.02	0.860 ± 0.02	0.372
PUFA/SFA	0.563 ± 0.01 ^d^	0.554 ± 0.00 ^e^	0.563 ± 0.01 ^de^	0.051
*ω*6/*ω*3	23.715 ± 1.19	25.456 ± 0.80	24.494 ± 1.65	0.108

I—control group, no additive; II—group with dried bovine colostrum; III—group with sodium butyrate; SFA—saturated fatty acid; MUFA—monounsaturated fatty acid; PUFA—polyunsaturated fatty acid. ^a,b^—Values within a row with different superscripts differ significantly at *p* < 0.05. ^d,e^—Values in the row with different superscripts show a trend 0.05 < *p* < 0.1.

**Table 4:** Effect of colostrum or sodium butyrate on the results of concentrations of selected mineral elements.

	Nutrition Group				
	I	II	III	SEM	*p*-Value
Number of piglets, no	6	6	6		
Mg	178.981 ^a^	192.454 ^b^	176.693 ^a^	2.03	0.0001
Ca	38.641 ^a^	43.315 ^b^	44.616 ^b^	0.91	0.009
Na	629.852 ^a^	652.717 ^ab^	689.986 ^b^	9.90	0.032
K	3188.933 ^a^	3421.183 ^b^	3224.917 ^a^	35.02	0.006
Zn	172.232 ^ab^	157.770 ^a^	200.431 ^b^	7.97	0.076
Cu	15.100 ^a^	28.269 ^b^	30.103 ^b^	2.15	0.002
Fe	199.923 ^a^	243.476 ^b^	171.462 ^c^	8.31	0.0001
Mn	2.309	2.455	2.013	0.07	0.041
Cr	0.428	0.450	0.465	0.01	0.170
Pb	0.236	0.243	0.267	0.01	0.411
Cd	0.026	0.023	0.026	0.001	0.187
Se	0.626	0.635	0.639	0.02	0.960
P	3348.522	3608.594	3195.775	50.23	0.0002
Al	0.586	0.733	0.778	0.04	0.149

I—control group, no additive; II—group with dried bovine colostrum; III—group with sodium butyrate. ^a,b,c^—Values within a row with different superscripts differ significantly at *p* < 0.05. SEM: error of the mean.

**Table 5:** Results of genomic analysis (qPCR) of piglet liver.

	RQ			
	Mean ± SD			
	I	II	III	*p*-Value
Number of piglets, no	6	6	6	
*ACADM*	0.890 ± 0.17	1.060 ± 0.24	1.004 ± 0.14	0.386
*CAT*	0.726 ± 0.31	0.888 ± 0.34	0.786 ± 0.11	0.520
*PPAR*	1.011 ± 0.11 ^a^	0.824 ± 0.19 ^ab^	0.600 ± 0.10 ^b^	0.006
*TNF*	0.855 ± 0.20 ^de^	0.910 ± 0.22 ^d^	0.670 ± 0.11 ^e^	0.082
*NOTCH1*	0.908 ± 0.08	1.102 ± 0.33	0.978 ± 0.28	0.676
*SIRT3*	1.096 ± 0.30 ^d^	0.893 ± 0.14 ^de^	0.796 ± 0.12 ^e^	0.082
*SOD2*	1.052 ± 0.39	0.713 ± 0.14	0.703 ± 0.27	0.318
*GSS*	1.037 ± 0.10	1.139 ± 0.39	0.971 ± 0.20	0.645

I—control group, no additive; II—group with dried bovine colostrum; III—group with sodium butyrate. ACADM—acyl-coenzyme A dehydrogenase, CAT—catalase, PPRA—peroxisome proliferator-activated receptor alpha, TNF—tumor necrosis factor α, NOTCH1—Notch Receptor 1, SIRT3—Sirtuin 3, SOD2—Superoxide Dismutase 2, GSS—Glutathione synthetase. ^a,b^—Values within a row with different superscripts differ significantly at *p* < 0.05, ^d,e^—Values in the row with different superscripts show a trend 0.05 < *p* < 0.1.

**Table 6:** Effect of colostrum or sodium butyrate on the results of the average content of short-chain fatty acids (SCFAs) in the large intestine content of piglets at 56 days of age (µmol/g wet weight).

	Nutrition Group				
	I	II	III	SEM	*p*-Value
Number of piglets, no	6	6	6		
Fatty acids (SCFAs)					
Acetic	63.10 ^d^	70.04 ^d,e^	76.02 ^e^	1.15	0.003
Propionic	32.80	34.27	37.18	0.98	0.258
Butyric	15.15 ^b^	20.18 ^a,b^	22.98 ^a^	0.64	0.014
Isobutyric	0.75 ^b^	1.14 ^a,b^	2.10 ^a^	0.13	0.024
Valeric	2.01 ^b^	2.88 ^a,b^	2.78 ^a^	0.12	0.038
Isovaleric	0.79	0.88	1.15	0.09	0.052
Sum of SCFA (µmol/g wet weight)	114.60 ^b^	129.38	140.21 ^a^	11.7	0.032

I—control group, no additive; II—group with dried bovine colostrum; III—group with sodium butyrate. ^a,b^—Values within a row with different superscripts differ significantly at *p* < 0.05. ^d,e^—Values in the row with different superscripts show a trend 0.05 < *p* < 0.1. SEM: error of the mean.

## Data Availability

Mendeley Data, V1, doi: 10.17632/zknw36f4rf.1 (30 May 206).

## Supplementary Materials

The following supporting information can be downloaded at: https://www.mdpi.com/article/10.3390/nu18111784/s1. Table S1: Composition of compound feeds. Figure S1: Representative microscopic images of the liver, pancreas, and kidneys. Figure S2: Alpha diversity comparison across I-control, II, and III groups. Figure S3: Principal coordinates analysis (PCoA) of Bray–Curtis dissimilarity with PERMANOVA results. Figure S4: Manhattan plot of relative abundance differences in bacterial families.
