# Supplementary material for: The Influence of Bovine Colostrum and Sodium Butyrate Supplementation on Gut Microbiota and the Intestinal–Liver Axis in Weaned Piglets
Source: Nutrients. 2026 Jun 1;18(11):1784. doi: 10.3390/nu18111784 (PMC13258546; doi:10.3390/nu18111784)
Supplement: Supplementary file 1 [file nutrients-18-01784-s001.zip › nutrients-4181453-supplementary.pdf]

Supplementary

The influence of the addition of cow colostrum or sodium butyrate to the diet of weaned piglets on the regulation of microflora and energy metabolism of the intestinal-liver axis

Pieszka, M.[1], Szczepanik, K.[1*], Lepczyński, A.[2], Marynowska, M.[2], Oczkowicz, M.[3], Ożgo , M.[2], Łoniewski, I.[4], Orczewska-Dudek, S.[1], Adaszyńska-Skwirzyńska, M.[5], Śliwiński B.[1], Skonieczna-Żydecka, K.[4]

[1] National Research Institute of Animal Production, Department of Animal Nutrition and Feed Sciences, Krakowska Str. 1, 32-083 Balice, Poland; marek.pieszka@iz.edu.pl, kinga.szczepanik@iz.edu.pl, sylwia.orczewska@iz.edu.pl, bogdan.sliwinski@iz.edu.pl

[2] Department of Physiology, Cytobiology and Proteomics; Faculty of Biotechnology and Animal Sciences, West Pomeranian University of Technology in Szczecin, Janickiego 29, 71-270 Szczecin, Poland, malgorzata.ozgo@zut.edu.pl, adam.lepczynski@zut.edu.pl, marta.marynowska@zut.edu.pl

[3] Department of Animal Molecular Biology, Krakowska Str. 1, 32-083 Balice, Poland; maria.oczkowicz@iz.edu.pl

[4] Sanprobi sp. z o.o. sp. k., Kurza Stopka 5/C, 70-535 Szczecin, Poland. Department of Biochemical Science, Pomeranian Medical University in Szczecin, 71-460 Szczecin, Poland; karolina.skonieczna.zydecka@pum.edu.pl, sanprobi@sanprobi.pl

[5] Department of Monogastric Animal Sciences; Faculty of Biotechnology and Animal Sciences, West Pomeranian University of Technology in Szczecin, Janickiego 29, 71-270 Szczecin, Poland, michalina.adaszynska@zut.edu.pl

* Correspondence: kinga.szczepanik@iz.edu.pl, +48666081411

Supplementary Table S1

Composition of compound feeds.

Composition of weaning feed	Prestarter	Starter
Barley, %	22,7	22,0
Wheat, %	20,0	27,0
Corn, %	15,0	20,0
Post-extraction soybean meal, %	25,0	21,0
Casein, %	9,0	4,50
Soybean oil, %	3,0	2,0
Forage chalk, %	0,8	0,80
1-Ca Phosphate, %	1,10	1,10
Feed salt, %	0,18	0,20
L-lysine, %	0,40	0,48

DL-methionine, %	0,15	0,20
L-threonine, %	0,12	0,15
L-Tryptophan 98 %	0,05	0,07
Premix[1], %	0,5	0,5
Nutrient content per kg DM:		
Metabolic energy, MJ	14,2	13,8
Crude fiber, g	30	40
Crude protein, g	200	180
Crude fat, g	47,5	42,8
Lysine, g	13,0	12,0
Methionine+cystine, g	6,6	6,5
Threonine, g	8,0	7,6
Tryptophan, g	2,5	2,2
Calcium, g	7,6	7,6
Phosphorus, g	6,5	6,0
Sodium, g	1,6	2,0

[1]Composition of LNB's mineral and vitamin premix in 1 kg: Na - 50 g, Ca - 208 g, P - 42 g; Mg - 10 g; Lysine - 60 g; Methionine - 12 g; Valine - 10 g, Threonine - 21 g, Vitamin A - 380000 IU; Vitamin D3 - 50000 IU; Vitamin E - 3500 mg; Vitamin K3 - 125 mg; Vitamin B1 - 57 mg; Vitamin B2 - 152 mg; Vitamin B3 - 1000 mg; Vitamin B6 - 114 mg; Vitamin B12 - 1.2 mg; Niacin - 1000 mg; Folic acid - 125 mg; Biotin - 7.5 mg; Fe - 3300 mg; Mn - 1330 mg; I - 50 mg; Zn - 3010 mg; Cu - 510 mg; Co - 40 mg; Se - 12 mg.

Supplementary Figure S1

Representative microscopic images of the liver, pancreas, and kidneys.

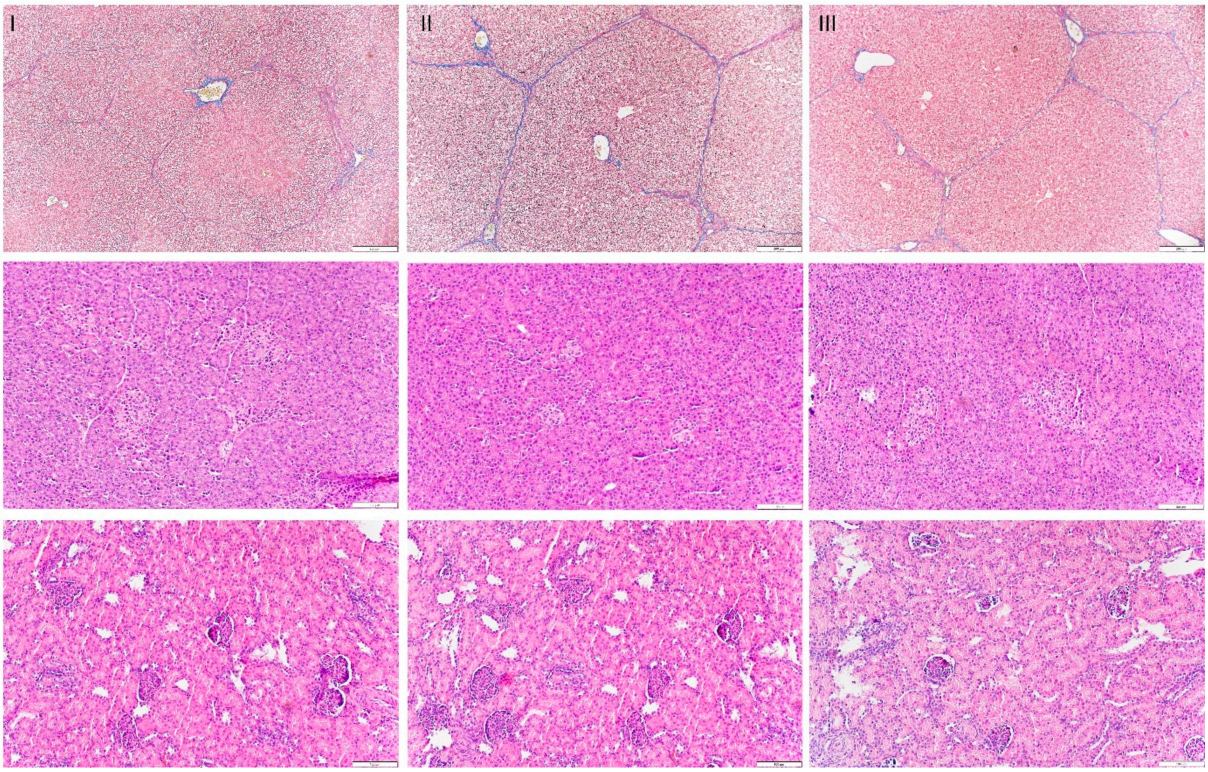

The columns represent groups (I - control group - no additive, II – group with dried bovine colostrum, III - group with sodium butyrate), while the rows represent, in order: liver (Masson trichrome staining with aniline blue), pancreas, and kidneys (H&E staining). Scale marked on the images.

Supplementary Figure S2

Alpha diversity comparison across I-control, II, and III groups.

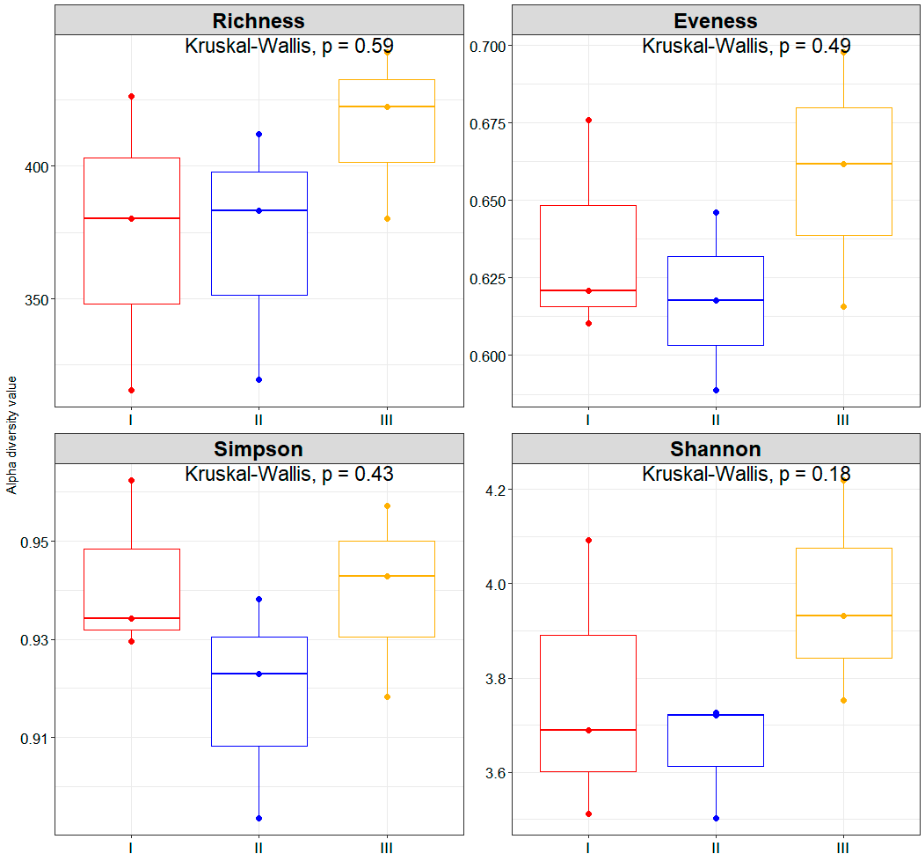

A boxplot was constructed to visually represent the distribution of four alpha diversity metrics—Richness, Evenness, Shannon, and Simpson—across three distinct experimental groups: I - control group - no additive, II – group with dried bovine colostrum, III - group with sodium butyrate. Each boxplot is accompanied by labels denoting the results of the Kruskal-Wallis test, with the p-value serving as a measure of statistical significance. Alpha diversity comparison across I-control, II, and III groups. Each group included 6 animals, from which 2 fecal samples per animal were collected and analyzed (n = 12 samples per group).

Supplementary Figure S3

Principal coordinates analysis (PCOa) of Bray-Curtis dissimilarity with PERMANOVA results.

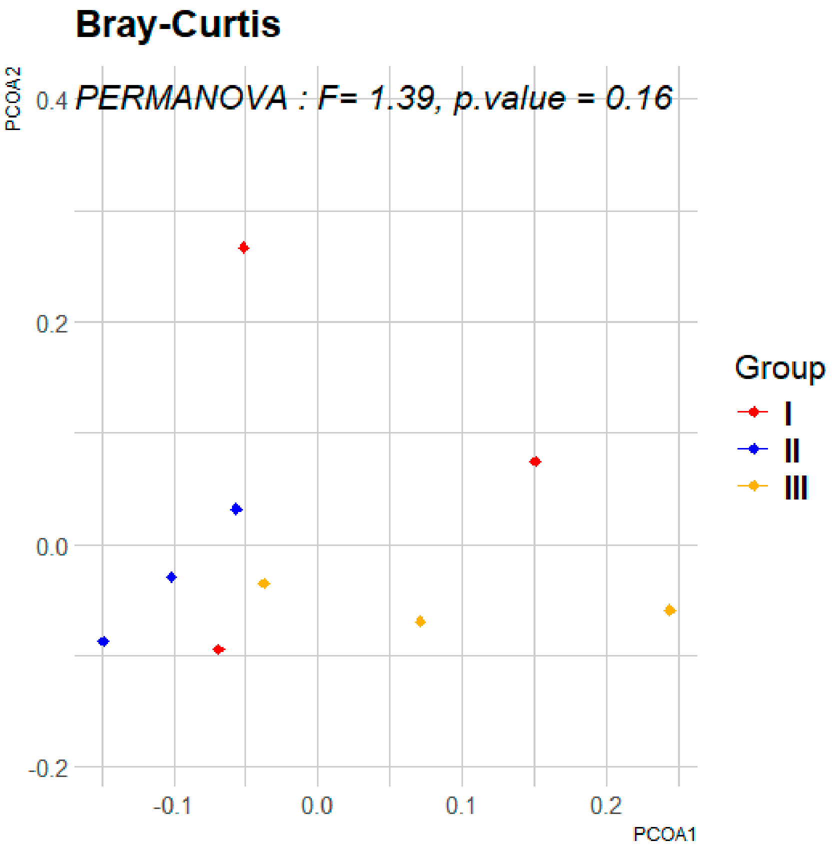

A Principal Coordinates Analysis (PCoA) was conducted to elucidate the underlying patterns of microbial community structure based on Bray–Curtis beta diversity metrics across the three experimental groups: I, II, and III. Each experimental group included 6 animals, from which 2 fecal samples per animal were collected and analyzed (n = 12 samples per group). Each data point on the PCoA plot represents a sample, and the distances between points reflect the dissimilarities in microbial community composition. Labels on the plot denote the results of the PERMANOVA test, including F-statistic values and associated p-values. The PERMANOVA test was selected for its robustness in analyzing multivariate data and assessing differences in group centroids while accounting for potential confounding factors.

Supplementary Figure S4

Manhattan plot of relative abundance differences in bacterial families.

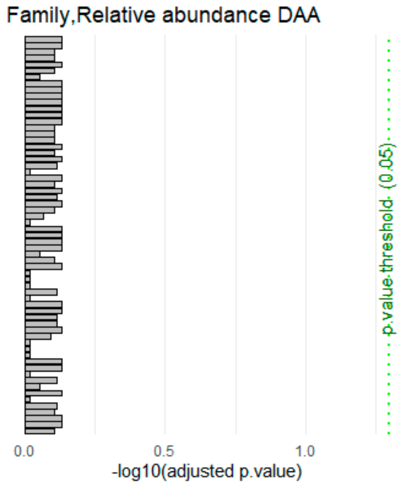

Differential abundance analysis was performed using rarefied abundance data transformed into relative abundances. Mean values across the study groups were compared using the Kruskal–Wallis test. Each experimental group included 6 animals, from which 2 fecal samples per animal were collected and analyzed (n = 12 samples per group). The results were visualized using a Manhattan plot displaying adjusted −log10(p-values) against the analyzed features. The significance threshold is indicated in green.
